# Review

**Published:** 1869-11

**Authors:** J. G. Willis


### REVIEW.

BY J. G. WILLIS, M. D.

Every member of the Dental profession who desires to be classed among the learners in the science and art of Dentistry must have an honorable pride in the rapid advancement made by the profession in the last few years.

This onward and upward movement is largely due to the various Dental associations throughout the United States, each vieing with all the others, to show the most substantial progress in not only that particular knowledge useful to Dentists, or specialists, but also in all departments of science; a knowledge of which is necessary to all persons before they can consider themselves learned. The influence these associations exert on the profession at large for good is beyond calculation.

Among them all, however, the American Dental Association stands pre-eminent, and the report of its proceedings is watched for eagerly by such as are not permitted to attend its annual sessions, for the reason that it is considered in the light of a Supreme Court of Dentistry, in which are tried, by the most rigid scrutiny, all new theories advanced, and all new methods of practice are by it thoroughly investigated, rejecting of the former, such as are not supported by facts, and accurately weighing the merits of the latter by the statistics of success and failure. A judgment of a court of last resort is final, right or wrong until reversed by the same ultimate authority.

The learned body of which I am speaking does not, I think, claim an authority quite so arbitrary as that. Therefore, while its opinions are entitled to great weight, they are also legitimately open to criticism. From the manner in which the business of the Association is conducted, it must be held responsible for every proposition introduced, that is received without objection, no matter how erroneous the same may be. It becomes the duty, therefore, of every member of the profession, to see to it, that he carefully reads the published report of proceedings, and if he discovers what he believes to be unsound doctrine, or a proposition contrary to facts, it is his bounden duty, through the journals to prove the doctrine unsound, or the proposition untrue. There are several vulnerable points in the proceedings as published, but I will only at this time consider one proposition—made and repeated—which I believe to be untrue.

It was stated by a member, as eminent for his skill in Dentistry, as for his attainments in general science. That the compound generally known as oxy-chloride of zinc, was in fact a hydrochlorate of zinc. I am not more surprised at his making the statement, than that, among all the savants of the profession there assembled, there was not found one bold enough to doubt the truth of the statement. The author of the same may, therefore, consider himself as an authority whose correctness it is temerity to question.

There were some very pointed remarks made by an eminent member, as to the wonderful ignorance of mankind at large on the subject of chemistry. I do not think that he meant to include himself and fellow-members in that comprehensive term, "mankind at large;" but their remissness in letting a statement in opposition to generally accepted facts, pass unnoticed is open to two explanations, which every person may make for himself.

Oxy-chloride of zinc is a compound occasionally used for filling teeth, and is made by adding to an oxide of zinc hydrochloric acid which has been saturated with zinc. The question for us to consider then, is whether it is properly called oxy-chloride of zinc. I propose to answer this question, making use of symbols which all persons at all acquainted with chemistry will understand.

It is a fact in chemistry, that all acids when not themselves decomposed unite only with the oxides of their several bases. Thus sulphate of soda is $SO_3NaO$, if it were otherwise the compound would be a sulphate of sod*ium*, and would be indicated by $SO_3Na$. The proper termination of this class of compounds according to the accepted chemical terminology, is *ate*, as phosph*ate*, carbon*ate*, muri*ate* (or hydrochlor*ate*). Hydrochloric acid is composed of hydrogen and chlorine, and is thus expressed by symbols, HCl. This acid will not unite with zinc in any form, without being decomposed, as is readily shown, thus, HCl added to pure zinc (Zn) produces a *chloride* of zinc and hydrogen is liberated. Thus $HCl+Zn=ClZn+H$, the latter being free of course escapes. HCl then is actually a chloride of hydrogen, and, as in the above experiment, zinc is substituted for the hydrogen, and the latter is set free, the compound can only be a chloride of zinc. But the same decomposition of the acid takes place when the *oxyd* of zinc is used as a base. $HCl+ZnO=ClZnO+H$ the hydrogen in this case also by a decomposition of the acid being set free, and the other element of the acid uniting with the zinc forms the chloride of the oxide of zinc, or oxy-chloride of zinc. The reason of the decomposition of the acid in these experiments is because chlorine has a stronger affinity for zinc than for hydrogen. That the above changes are different from those where hydrochlorates are formed, is readily seen, thus $HCl+AmO=HClAmO$, which is hydrochlor*ate* of ammonia. In this case, there is a direct union between the acid and the base without decomposition of the acid.

That the reaction between HCl and ZnO is correctly stated is abundantly proved by the fact, that to procure hydrogen in large quantities HCl and Zn are constantly used. Sometimes, however, *sulphuric* acid is used instead of HCl, and it may be instructive to notice the difference in the reaction which takes place when it is thus used, all other things being the same. Sulphuric acid is represented thus, $SO_3HO$ and the reaction is as follows viz: $SO_3HO+Zn=SO_3ZnO+H$, the latter (hydrogen) being set free. These changes are explained thus: the *water* (HO) of the acid is decomposed, the oxygen uniting with the zinc forming ZnO with which the acid unites, and leaving, as above stated, $SO_3ZnO$ and free hydrogen. $SO_3ZnO$ is the sulph*ate* of zinc, the hydrogen having been obtained in this experiment from the *water* of the acid without any decomposition of the acid itself.

It is unnecessary to pursue the subject further, as what has already been said proves conclusively that in accordance with the nomenclature of chemistry, the compound under consideration *is properly* called oxy-chloride of zinc. It has been said "that what comes out of the mouth, and not what goes in," defiles the soul; and herein it differs from chemistry, as only such elements as remain *in* a compound, have any influence in determining its physical characteristics; such as are given off during the reaction, have no effect on the compound whatever. And as the hydrogen of the hydrochloric acid is given off and set free, the compound can not be correctly called a *Hydro*chlorate, and as only *chloride* of zinc is left to unite with the oxyd of zinc, it can not correctly be called a chlor*ate*, as this latter is always formed by a union between chlo*ric acid* and some base, as $ClO_5+HO=ClO_5KO$=chlor*ate* of potash.

